# Twitter use in physics conferences

**DOI:** 10.1007/s11192-016-2031-1

**Published:** 2016-06-27

**Authors:** Stephen Webb

**Affiliations:** DCQE, University of Portsmouth, St Michael’s Road, Portsmouth, PO1 2PR UK

**Keywords:** Twitter, Scholarly communication, Subdisciplinary differences, Physics conference activity

## Abstract

An analysis of Twitter use in 116 conferences suggests that the service is used more extensively at PACS10 conferences (those devoted to the physics of elementary particles and fields) and PACS90 conferences (those devoted to geophysics, astronomy, and astrophysics) than at conferences in other fields of physics. Furthermore, Twitter is used in a qualitatively different manner. A possible reason for these differences is discussed.

## Introduction

I currently follow 680 Twitter accounts. As a reflection of my professional interests, many of these accounts belong to academics working in the fields of particle physics and astrophysics. My Twitter timeline is thus unsurprisingly dominated by tweets that might be termed “astroparticle” in nature. However, the increasing move towards collaborative research straddling the boundaries between traditional disciplines (Basner et al. 2013), combined with the phenomenon of retweeting (explained in “The Twitter platform” section below), gives rise to the surmise that my timeline should contain a certain proportion of tweets from physicists in other subdisciplines. This perhaps naïve expectation runs counter to my experience. Furthermore, in a (Twitter-mediated) conversation, the question was posed whether, during academic conferences, Twitter registered similar levels of use by physicists working in different subdisciplines. The stimulus for the question was the observation of low Twitter activity at an international conference on magnetism: anecdotal evidence suggested that members of the astronomy community, for example, were more active users of Twitter than members of the condensed matter community. A literature review failed to uncover research into this question. Therefore, in an attempt to determine whether there are subdisciplinary differences in Twitter use, I undertook a study of 123 scientific conferences whose theme was related to some area of physics or astronomy. Seven conferences were subsequently deemed unsuitable for analysis. An analysis of 8774 tweets from the remaining 116 conferences is consistent with the suggestion that different physics subdisciplines do indeed use Twitter in differing degrees.

This paper is organized as follows. For readers who are unfamiliar with what is still a relatively new tool for scholarly communication, the “Background and nomenclature” section presents some brief background to Twitter and explains the relevant nomenclature. The “Methods” section describes the methods used and the “Results” section presents the results of an analysis of the data. After the “Conclusion” section, in which possible reasons for the observed difference in Twitter use are explored, an “Appendix” lists the names of the conferences used in the analysis; this, combined with the open nature of Twitter, permits replication of the work.

## Background and nomenclature

### The Twitter platform

Twitter is an online microblogging platform that allows its users (“tweeps”) to publish messages of 140 characters or less. These messages (“tweets”) can also include URLs, images or videos. One user can choose to “follow” another; when someone publishes a tweet that message immediately appears in the timeline of all those who follow that person.

The 140-character limit essentially guarantees the absence of nuance in tweets, and there is a possibility that complex ideas might be reduced and misrepresented as “sound bites”. Nevertheless, Twitter’s combination of brevity and immediacy has made it a popular platform: as of June 2015 there were 316 million active users per month, with 500 million tweets being published each day (about.twitter.com 2015). Despite the message length restriction, tweeps use the service to publish opinions, gossip and news. In addition to its widespread application in politics and journalism, large-scale studies of Twitter use have been employed by scientists for a variety of ends, including the augmentation of earthquake response systems (Earle et al. 2011), the investigation of how health information is disseminated (Scanfield et al. 2010) and as a means of estimating crowd size (Botta et al. 2015).

A number of features enhance Twitter’s use as a communication tool and also facilitate subsequent analysis of tweets.

First, Twitter has a “retweet” function. If someone retweets a tweet, the original tweet appears in the timelines of that tweep’s followers and is identified as a retweet (RT). Holmberg and Thelwall (2014) identify a variety of reasons why people retweet, including: to spread information to a wider group; to publicly agree, or perhaps take issue with, someone’s opinion; to give visibility to unpopular content; in the hope of gaining reciprocity; and as an archival mechanism. Second, the use of @ followed by a username allows a tweep to send a message to another tweep, or to let @username know that he or she has been mentioned in a tweet. This affords a conversational aspect to the service (Boyd et al. 2010). Third, the use of the hashtag symbol # directly before a word permits a rudimentary but nevertheless effective form of tagging. Furthermore, it is possible to monitor hashtags in real time thus expediting access to tweets of particular relevance.

### Twitter use in scholarly scientific communication

The development of social media platforms has transformed the way in which people in general and scientists in particular communicate their ideas. Traditional communication channels, such as books, journals and broadcast media, involve a one-way flow of information. Social media platforms, which have allowed online communities, networks and crowdsourcing to flourish, encourage the interactive sharing of ideas. A growing number of social media platforms, including collaborative spaces, blogs, online content communities and professional networking sites, are not only supporting the scientific and scholarly enterprise but are also increasing the pace at which knowledge is developed and shared (see, for example, Bar-Ilan et al. 2012; Bik and Goldstein 2013).

A number of studies have attempted to understand the motivation behind scientists’ use of social networking sites in general and Twitter in particular. Williams et al. (2013) investigated Twitter use in the medical professions and identified a number of different applications of the service. Darling et al. (2013), while investigating the role of Twitter in the life cycle of a traditional research publication in a bioscience discipline, found that in a sample of 116 scientists the average number of Twitter followers (median 241) was seven times larger than the size of the average academic department (median 33). Even allowing for the fact that not all people who follow a scientist on Twitter are themselves scientists, Darling et al.’s work confirms that a virtual network can be substantially larger than the more traditional type of network. This can be a powerful motivating factor for scientists; furthermore, scientists can grow their virtual network with relatively little investment in time. A *Nature* survey (Van Noorden 2014) queried academics about their reasons for using social networking sites. Of 330 Twitter users who responded to the survey, the main reasons given for using the platform were to follow discussions, to post work content, to discover peers, to learn about recommended papers, and to comment on research.

One prevalent use of Twitter amongst scientists involves conference activity: at many conferences a number of delegates choose to tweet live from the event. The use of Twitter at conferences has received some attention in earlier work, but studies have been small-scale and focused on data from two or three conferences (e.g. Ross et al. 2010; Letierce et al. 2010; Weller and Puschmann 2011; Weller et al. 2011). Furthermore, the four studies referred to above involved conferences in Humanities (Ross et al. 2010) or Computer Science (Letierce et al. 2010; Weller and Puschmann 2011; Weller et al. 2011). A literature search failed to find similar studies in the context of physics conferences.

Few if any large-scale studies have examined scientists’ motivation for live tweeting at conferences. However, simply from reading conference tweets, one can posit a number of reasons. There is often a clearly scientific aspect to a tweet: a user might link to a preprint, for example. There can be an outreach aspect to a tweet: an individual can use Twitter to reach networks outside of academe. A conference participant might tweet in order to provide information, and a flavour of the event, to colleagues who cannot attend. An increasingly popular activity at scientific and technical conferences is a style of visual notetaking known as sketchnoting; some participants choose to share their notes using a #sketchnote hashtag. A tweet can be a means of raising points of contention; for example, the hashtag #everydaysexism has been used to flag conference speakers’ language or attitudes. A tweet can have a purely social aspect. Twitter can be used for networking: employers can tweet the availability of jobs and postgraduates can enquire about possible postdoctoral positions. Twitter can be used by conference organisers as a channel for news, and delegates can use the service to ask for information. The platform can be used by sponsors and exhibitors to advertise their presence, and by local authorities to extol the virtues of the area surrounding the conference venue. A qualitative study would surely determine yet more reasons why live tweeting takes place at scientific conferences.

The etiquette surrounding the use of Twitter at scientific conferences is still evolving. For example, at the annual meeting of the Ecological Society of America held in August 2015, the conference organisers’ request for attendees to gain consent from speakers before tweeting about presentations caused confusion (Woolston 2015). There remains debate about whether conference policy should default to allow live tweeting unless a speaker explicitly asks attendees not to do so. On the other hand, many conference organisers explicitly encourage delegates to tweet using a particular hashtag.

### Harvesting tweets for analysis

Searching historical Twitter data is possible, but can be costly if one wishes to perform anything other than basic analytics. However, Twitter makes publicly available APIs that allow one to access its network stream in two different ways. The first method allows a user, in real time, to harvest tweets that meet given conditions; thus a tweet containing a particular hashtag can be piped to a file for later analysis. The second method permits a user to search for and store all tweets posted in a preceding 7-day period that meet given conditions. These APIs gave sufficient access to the service for the purposes described in this paper.

## Methods

### Identification and classification of conferences

During summer 2015, a register of forthcoming academic conferences relating to subject areas in physics, astronomy and cognate disciplines was prepared from public listings on the web pages of major national physical societies, scientific publishers, and commercial conference management systems. A conference was deemed suitable for further study if it possessed (1) a well defined web presence in English and (2) a clearly identifiable hashtag or keyword that delegates might reasonably employ for live tweeting. In total, 123 conferences were identified.

The conferences were classified according to the Physics and Astronomy Classification Scheme (PACS) as developed by the American Institute of Physics (AIP) and used by the journal *Physical Review* since 1975. PACS is a hierarchical segregation of the entire spectrum of subject matter in physics, astronomy and certain related fields. The scheme contains ten broad subject categories, as shown in Table 1, but with a sub-hierarchy that includes at least four levels of depth; the narrowest term in the hierarchy provides the most detailed characterization of subject matter. Progress in indexing and searching technologies has highlighted various limitations of a scheme such as PACS, and in 2015 a new taxonomy for physics was introduced: the AIP Thesaurus replaces PACS and will aid in the retrieval of scientific information. Nevertheless, for the purposes of the present study, it was concluded that the top-level categorization of PACS as defined in the 2010 update was sufficient.

**Table 1:** Top-level headings of PACS 2010^®^

Classification	Branch of physics or astronomy
00	General
10	The physics of elementary particles and fields
20	Nuclear physics
30	Atomic and molecular physics
40	Electromagnetism, optics, acoustics, heat transfer, classical mechanics, and fluid dynamics
50	Physics of gases, plasmas, and electric discharges
60	Condensed matter: structural, mechanical, and thermal properties
70	Condensed matter: electronic structure, electrical, magnetic, and optical properties
80	Interdisciplinary physics and related areas of science and technology
90	Geophysics, astronomy, and astrophysics

Since my familiarity with physics is not equally spread across all branches of the subject, mis-classification of the primary conference theme was possible. However, this represented an unlikely source of error since only the broadest level of the scheme was used. For example, in December 2015 a workshop (which is not part of this analysis) was held on the topic “Modern nuclear density functional theory and its applications”. Subject-specific knowledge might be required to apply a narrow level of classification for this workshop, which would be 21.60 Jz. Much less specialist knowledge is needed in order to classify the conference under the generic heading PACS20 which, as Table 1 shows, refers to the very broad category of “Nuclear Physics”. Much of the analysis in this paper hinges simply upon judging if the primary classification of a conference is PACS 10 or PACS 90, or else if it is associated instead with one of the other eight PACS numbers.

A note on nomenclature: in this paper, a conference classified as PACS 10 or PACS 90 will be denoted as an “Astro/Particle” conference. The term thus refers to a conference that involved any aspect of the physics of elementary particles and fields (PACS 10) or of geophysics, astronomy, and astrophysics (PACS 90). The term “Astro/Particle” should not be confused with the emerging subdiscipline of astroparticle physics. Any conference not in PACS 10 or PACS 90 is grouped in the term “Other(s)”.

### Conferences included in the study

Of the 123 conferences identified in the “Identification and classification of conferences” section, seven were rejected for further analysis. Three of these seven were rejected because, although physicists were invited to participate, later examination showed that the focus of the conference lay outside the classification scheme being used. (Two conferences had their focus on scientific computing, the other solely on chemistry). A further three conferences were rejected because it proved impossible to obtain more details about them and thus classify them with confidence. These three conferences were all based in China. No Twitter activity was associated with them, but whether this was due to firewall restrictions or simple lack of use of the service is not known. A final conference was rejected for perhaps more interesting reasons. A conference devoted to Hawking radiation attracted 32 participants but during the event 139 different Twitter accounts used the conference hashtag. Further investigation showed that four of the 32 participants possess Twitter accounts, but none of them tweeted using the conference hashtag (although one participant did tweet from the conference without using the hashtag). Upon inspection of tweet content and associated metadata it became clear that the conference hashtag was being used by people around the world, not necessarily scientists, who were interested in a public lecture given by Professor Stephen Hawking. Public outreach is certainly a significant aspect to Twitter use and, as discussed in the concluding section, the questions this raise are certainly worthy of further study. However, the use of Twitter at this particular scientific meeting was different in kind to other conferences in the study and it was therefore rejected for further analysis. The “Appendix” section contains the titles of the 116 conferences that were analysed for Twitter activity.

No attempt was made before taking data to identify equal numbers of conferences across each of the various subject areas. Table 2 gives the number of conferences in each classification and, as can be seen, 21 conferences were classified as “Astro/Particle” and 95 conferences were classified as “Other”.

**Table 2:** Number of conferences in each classification group

PACS classification	Classification in this paper	Number of conferences chosen for analysis
00	Other	26
10	Astro/Particle	7
20	Other	9
30	Other	5
40	Other	7
50	Other	4
60	Other	11
70	Other	12
80	Other	21
90	Astro/Particle	14

The 116 conferences selected for analysis covered a wide geographical spread, with venues situated in 34 different countries. Broadly, these were split into those taking place in UK/Europe (80 events), Asia/Australasia (19 events), US/Canada (14 events) and Latin America (3 events). The title of many conferences explicitly expressed an international flavour to the event (see “Appendix” section) but in all cases the language of the conference website was English.

Note was also taken of conference duration and the number of participants. The intention behind this was to obtain a better measure of Twitter activity than a simple count of the number of tweets: a 1000-delegate conference of five days’ duration has more available “space” for Twitter activity than a 50-delegate workshop lasting 2 days. Clearly, conference duration is a straightforward matter of record. The figures for participant numbers, however, should be treated with a degree of caution. In many cases, the conference website or other publically accessible channel published a list of names and affiliations of those registered to attend the conference; this gave a precise number of participants, and the number was checked both during and after the conference. Post-conference figures were used when available. In about the same number of cases, further research was required in order to ascertain participant numbers; the necessary information was available in post-conference reports, learned society publications, open source event-organising sites such as Indico, and so on. In a small number of cases, conference organisers were approached directly and asked for participant numbers. However, although precise figures for registered participants can be obtained, these are not necessarily completely accurate. For example, it is possible that some people found themselves unable to attend a conference but still appear on a list of delegates; others might have registered late and do not appear on such a list; yet others might have requested not to have their names appear on a public list. Furthermore, not all participants attend all available sessions of a conference. Nevertheless, although there is an inevitable uncertainty in the metric, the product of conference participants and conference duration does seem to provide a reasonable measure of the size of an event. Fortunately, as will be seen in “Results” section, an uncertainty in participant number as large as 10 % (which could be viewed as being unduly pessimistic) turns out not to affect the conclusions.

The conferences in this sample ranged in size from 1851 participants at one extreme to 40 participants at the other. An analysis showed no relationship between the number of participants and the level of twitter activity.

### Conference hashtags

Two programs were written to harvest tweets that contained a conference hashtag or related keyword: one program used a Search API, the other a Streaming API. The latter API keeps a persistent http connection open; the former requires the polling of a rest endpoint, but is perhaps better suited for singular searches. Twitter itself states that the Search API is not a complete index of all tweets, and research by Gonzalez-Bailon et al. (2014) shows that the Search API “over-represents the more central users and does not offer an accurate picture of peripheral activity”. Nevertheless, in initial tests aimed at harvesting conference-based tweets, the program based on the Search API was found to be more robust. In particular, for conferences where Twitter activity was low, the Streaming API harvested fewer tweets (due primarily to time-out issues) than the Search API. On the other hand, every tweet identified by the Streaming API was also identified by the Search API. Therefore, the decision was made to harvest conference tweets using the Search API.

Some conference delegates might choose to tweet from the event without using a hashtag or keyword, and such content—even if it were directly relevant to the conference—would be invisible to the harvesting program. By the same token, however, the same content would be difficult to identify for other users. It is likely, therefore, that the number of such untagged tweets will be small compared to the number labelled with a conference hashtag.

It should also be noted that there is degeneracy amongst hashtag usage: different conferences legitimately employ the same hashtag. In order to minimise the likelihood of harvesting inappropriate tweets, the search on a particular hashtag or keyword was limited to the dates on which the relevant conference was held. For example, the hashtag #isb2015 was used by a conference organised by the International Society of Biomechanics but also for one organised by the International Society of Bassists (and quite possibly by several other societies); limiting the search to the dates on which conferences were held was a way of minimising this effect, at the expense of missing out on pre- and post-conference tweets. There were also occasions on which the use of a hashtag was contested during an event. For example, #pathways2015 would have been a legitimate hashtag for one of the most Twitter-active conferences in this sample, “Pathways Towards Habitable Planets—II”. Indeed, this particular hashtag was used early in the conference. However, the same hashtag was being used coterminously by a Bible camp. Participants at the exoplanets conference self-organised on Twitter and agreed to use the hashtag #pathwaysII instead. This self-organising behaviour was observed on several occasions in this sample. (In one plaintive occurrence a nuclear physics conference was the source of two tweets in total, both from the same physicist, who discussed with him/herself an appropriate hashtag for the event. No one else tweeted from the conference, using those or other hashtags). In some cases interference was observed between an agreed conference hashtag and its use by a wider community for other purposes. For example, the hashtag chosen for a conference on materials chemistry, #MC12, is used by people interested in a particular make of Maserati sports car. In such cases, irrelevant tweets had to be removed by hand before further analysis was undertaken.

## Results

### Twitter activity at conferences

Over the period 12 July 2015 to 2 October 2015, Twitter usage was captured from the 123 initially identified conferences, and data was analysed from the 116 conferences discussed in “Conferences included in the study” section. The 116 conferences were grouped into those classified as PACS10 or PACS90 (“Astro/Particle”) and those in all other PACS classifications (“Other”): 21 conferences were classified as Astro/Particle and 95 as Other. As mentioned above, an initial analysis showed no correlation between number of conference participants and level of Twitter activity.

Use of a conference hashtag was recorded at 72 of the 116 conferences in the sample. Interestingly, the 44 conferences that recorded zero Twitter activity were not proportionately distributed between the two groupings: only 2 of the 21 Astro/Particle conferences registered zero Twitter activity whereas 42 of the 95 Other conferences made no use of Twitter.

If these conferences form a representative sample then the measurement above is consistent with one of the anecdotal observations that prompted this study: a delegate at an Astro/Particle conference has only a 10 % chance of attending an event exhibiting zero Twitter activity whereas a delegate at Other conferences has a 44 % chance of attending an event in which Twitter is not used. In other words, a delegate at a non-Astro/Particle conference is about four times more likely to be participating in an event with a zero level of Twitter activity than a delegate at an Astro/Particle conference.

In order to explore conference Twitter use in more detail, events with zero Twitter activity were excluded from subsequent analysis. Table 3 shows data for the 72 conferences that registered at least one tweet.

**Table 3:** Data from 72 tweeted conferences, grouped into those classified as PACS10/PACS90 (Astro/Particle) and those in all other PACS classifications (others)

	Astro/Particle	Others (all)	Others (most active)
No. of conferences	19	53	19
No. of conferences with <5 tweets	1	15	0
No. of conference tweets (incl. retweets)	5941	2833	2617
No. of conference tweets (excl. retweets)	2596	1569	1347
No. of tweeps	1316	785	653
No. of originating tweeps	246	406	326
Total number of conference days	85	269	101
Total number of conference delegates	3927	28,582	13,681
Tweeps as a % of delegates	33.5	2.7	4.8
Originating tweeps as a % of delegates	6.3	1.4	2.4

For the purposes of comparison, the final column contains data from the 19 conferences in the Others category that produced the most tweets. (The term “tweep” refers to a Twitter user who either posted a tweet containing the conference hashtag/keyword or who retweeted such a tweet. The term “originating tweep” refers to a Twitter user who posted at least one original message containing the hashtag, i.e. someone who posted something other than a retweet)

No attempt was made to estimate the total number of physics conferences that take place in a given time period, and so the data shown in Table 3 cannot be used to explain the anecdotal observation that tweets from Astro/Particle conferences outnumber those from Others. Nevertheless, the data are suggestive. Other conferences outnumbered Astro/Particle conferences in this sample by a factor of 2.8, but the latter generated 1.7 times as many original tweets as the former. Retweeting was widespread throughout, but a tweet at an Astro/Particle conference was more likely to be retweeted: on average, each tweet in the Astro/Particle grouping generated 1.3 retweets whereas in Others a tweet generated only 0.8 retweets. Overall, if retweets are included, there is a clear difference between Astro/Particle and other conferences: 2.8 times fewer conferences generated 2.1 times as many tweets.

If one defines low conference Twitter activity as being the production of fewer than five tweets (the mean conference duration was just under 5 days, so fewer than five tweets means on average less than one tweet per day containing the conference hashtag) then only one of the 19 Astro/Particle conferences (5 %) exhibited low levels of activity. Amongst Others, however, 15 of the 53 conferences (28 %) had low levels of Twitter activity.

The number of tweeps in the two groupings is also instructive. An estimate of those who tweeted live is given by those who generated at least one original tweet pertaining to a conference. (Non-attendees were observed to retweet tweets with a conference hashtag, but they are unlikely to post original tweets about a conference they are not attending). Table 3 shows that 6.3 % of Astro/Particle delegates (“originating tweeps”) chose to live tweet while the equivalent figure for Others was 1.4 %—a difference of a factor of 4.5. (Note that in both cases there might be an element of double counting since some delegates attend more than one conference. Furthermore, some delegates tweet under more than one account. The uncertainty on delegate numbers has already been mentioned. Nevertheless, in this dataset, live tweeting is clearly more prevalent at Astro/Particle conferences than Others. An uncertainty in delegate numbers at the level of 10 % does not alter the conclusion).

When retweeting is taken into account, the difference between the two groups is even more clear: 33.5 % of Astro/Particle delegate numbers posted at least one tweet or retweet while the equivalent figure for Rest was 2.7 %—a difference of a factor of 12.4. As noted above, those who retweeted were not necessarily conference delegates themselves and might have been retweeting a post that appeared in their timeline. However, this finding is consistent with the anecdotal observation mentioned in the introduction.

A better measure of conference Twitter activity is one that takes into account the available “space” for such activity. The average tweet rate is defined here as the number of tweets per delegate per conference day, multiplied by a factor of 1000 for ease of analysis. If retweets are included, the tweet rate for Astro/Particle is 17.8; for Others the tweet rate is 0.37. This measure thus exhibits a difference of a factor of 48 between the two groups.

In the above analysis we have chosen to ignore conferences with zero Twitter activity, a choice that overwhelmingly favours Others over Astro/Particle conferences. If we go even further and choose to consider only the 19 Other conferences that produced the most tweets, there remains a clear difference between Astro/Particle conferences and Others. As is illustrated in the final column in Table 3, the percentage of tweeps is greater for Astro/Particle, as is the percentage of those who live tweet. The tweet rate for Astro/Particle is a factor of 9.4 times greater than for Others (a tweet rate of 17.8 compared to 1.9).

The five most active Twitter conferences in terms of tweet rate were all PACS90 events: “ASB6: The Origin, Distribution and Detection of Life in the Universe” produced the largest tweet rate (6581, including retweets), followed by “Exomol 2015—Spectroscopy of Exoplanets” (1944), “Accurate Astrophysics. Correct Cosmology.” (842), “SDSS-IV Collaboration Meeting” (833), and “Pathways towards Habitable Planets II” (551). Excluding retweets does not change the conclusion: the same five conferences were the most active and the order is only slightly changed (the SDSS Meeting was more active than the Accurate Astrophysics conference in terms of live tweeting).

Table 4 shows the result of grouping the 72 conferences into those with the 12 most active tweet rates and those with the 12 least active tweet rates (each group thus corresponding to one-sixth of the total number of conferences); the remaining two-thirds are considered to exhibit a mid-range level of Twitter activity. As the table shows, all 12 least-active conferences were Others. Conversely, Astro/Particle conferences accounted for 9 of the 12 most-active conferences. Excluding conferences at which there was no Twitter activity, the median tweet rate for Astro/Particle conferences was $65 _{19}^{306}$ while for Others it was $8 _{3}^{14}$, where the sub and superscripts denote the 95 % confidence intervals on the median. If all 116 conferences in the sample are considered, the difference is even starker: the median tweet rate for Others falls to $1_{0}^{2}$ while the figure for Astro/Particle is essentially unchanged.

**Table 4:** The 72 conferences grouped into the sixth with least activity in terms of tweet rate, the sixth with most activity in terms of tweet rate, and the two-thirds that exhibit a mid-range level of activity

Level of activity	Astro/Particle	Others
Least activity (12 conferences, 16.7 % of total)	0	12
Mid-range activity (48 conferences, 66.7 % of total)	10	38
Most activity (12 conferences, 16.7 % of total)	9	3

Tweet rate = number of tweets per delegate per conference day, multiplied by a factor of 1000 for convenience

Two conferences, both of them Astro/Particle, generated more than 1500 tweets and retweets. “ASB6: The Origin, Distribution and Detection of Life in the Universe” (classified as PACS90) generated 1836 tweets and “EPS HEP 2015” (classified as PACS10) generated 1614 tweets. If retweets are excluded, the three conferences that produced the most tweets were all Astro/Particle: “ASB6: The Origin, Distribution and Detection of Life in the Universe” (857 tweets; PACS90); “SDSS-IV Collaboration Meeting” (415 tweets; PACD90); and “EPS HEP 2015” (346 tweets; PACS10).

Thus a variety of metrics suggest that Twitter activity is greater at Astro/Particle conferences than at those in other sub-branches of physics. It was suggested that a possible explanation for this observation might reside in conference location: perhaps the Astro/Particle conferences in the sample were more likely to be held in locations where Twitter is more heavily used. However, a location-based analysis disfavours this hypothesis. Liu et al. (2014) studied datasets of over 37 billion tweets in the period 2006–2013 in order to investigate how Twitter use had evolved. They showed that Twitter use has spread across the globe. Tweets from US/Canada constituted almost 80 % of the dataset when the service began, but by mid-2011 this had declined to 32 %—a figure that held relatively steady in the following years. The proportion of tweets from Europe was relatively stable from January 2011 onwards, at about 20 % of all tweets. Asia showed more volatility than Europe in Twitter use, but at December 2013 the proportion was just over 20 %. The proportion of all tweets from Latin America was, in December 2013, just under 20 %. If conference Twitter activity in summer 2015 followed the proportions at December 2013 as identified by Liu et al. (2014) then, given the geographical spread of conferences in the sample as mentioned in the “Conferences included in the study” section, one might expect the top 12 most active conferences to contain 8 European events, 2 Asian/Australasian events and 2 US/Canadian events. In fact, in terms of tweet rate, the 12 most active conferences were all European; the most active US-based conference came 25th in the list, behind three Asian/Australasian events and a conference based in Latin America. Furthermore, whereas for conferences in UK/Europe, Asia/Australasia and Latin America the number of events with Twitter activity exceeded those with no activity, in US/Canada the reverse was true: 10 of the 14 conferences in these locations exhibited no Twitter activity.

That discipline is more important than location is also demonstrated when one compares tweet rates for conferences within individual countries. For example, of the 10 UK-based conferences exhibiting Twitter activity, 4 were Astro/Particle events and 6 were Others; the 3 most active conferences in terms of tweet rate were all Astro/Particle. Of the 4 US-based conferences exhibiting Twitter activity, 2 were Astro/Particle and 2 were Others; the 2 most active conferences in terms of tweet rate were Astro/Particle. Of the 6 Spain-based conferences exhibiting Twitter activity, 2 were Astro/Particle and 4 were Others; the 2 most active conferences in terms of tweet rate were Astro/Particle.

**Fig. 1:**
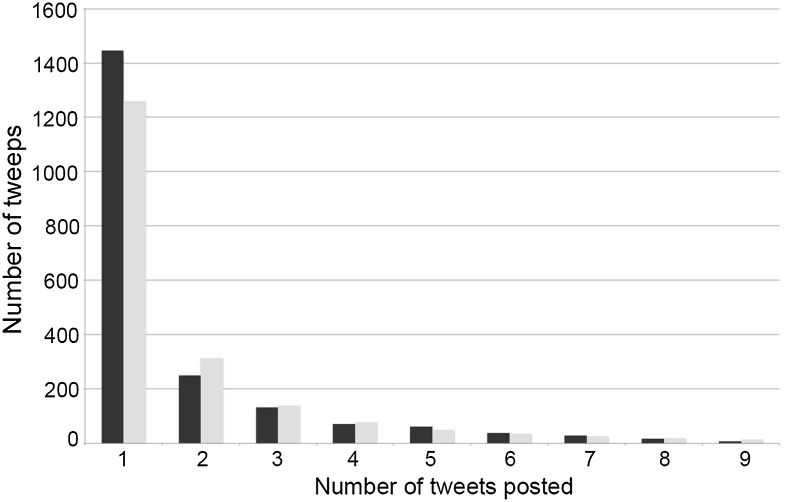
The number of tweeps posting between 1 and 9 tweets (including retweets and summing over all 77 conferences in the sample that exhibited some Twitter activity). The *dark bars* show the observed numbers. The *lighter* *bars* are the numbers generated by Eq. (1). As can be seen, the observations possess power law characteristics

An investigation of the pattern of Twitter usage at the conferences suggests that a different factor might be at play when considering the difference between Astro/Particle and Others. If the dataset is taken as a whole, including retweets and summing over all PACS disciplines, then the distribution of the number of tweets posted by each user has power law characteristics: many users posted only one tweet or retweet while a small number of users posted many tweets. Over the range from 1 to 9 tweets, the relationship between *T* (the number of distinct Twitter accounts) and *N* (the number of times a user tweeted) is shown graphically in Fig. 1 and is well approximated by the following equation:

1 $$\log_{10} T = 3.1 - 2\log_{10} N$$

Such power law behaviour is in line with other analyses of the use of Twitter in science (e.g. Letierce et al. 2010), and is a common characteristic of web-based phenomena. Beyond this region there are a number of black swans: individuals who make greatly more use of the service than most others. In this sample the tail extends as far as one user who posted 677 tweets in a 3-day conference. In all, the number of users who posted 10 or more tweets is roughly equal to the number who posted 3 tweets. However, whereas the 133 tweeps posting 3 tweets were responsible for 399 tweets in total, the 135 tweeps posting 10 or more tweets were responsible for 5182 tweets (59 % of the total number of tweets harvested during this study).

An examination of the 28 conferences at which someone posted 10 or more tweets containing the conference hashtag highlights a clear difference between Astro/Particle and Others. If one ignores conferences with no Twitter activity, then 63 % of Astro/Particle conferences (12 of 19) attracted at least one such highly Twitter-active account. The equivalent figure for Others was 30 % (16 of 53). Thus, where Twitter is used at all, Astro/Particle conferences are twice as likely as Others to have someone posting 10 or more tweets using the conference hashtag.

A total of 71 tweeps posted 10 or more tweets containing an Astro/Particle conference hashtag. (This figure of 71 accounts is equivalent to 1.8 % of the total number of delegates at Astro/Particle conferences where Twitter was used). For Others, 64 tweeps (equivalent to 0.22 % of the total number of delegates) posted 10 or more tweets. An analysis of tweet content and public user profiles was then used to determine whether these highly active users were in fact conference attendees or were simply retweeting content from a distance.

Of the 71 active Astro/Particle tweeps, 10 were individuals with an interest in science but who were not conference delegates; these 10 people were responsible for a total of 317 retweets. The remaining 61 highly active accounts were all conference attendees; this equates to 1.6 % of Astro/Particle conference delegates. Of these 61 accounts, 16 were organizational in nature (conference organisers, collaborative experiments, and so on); 45 accounts belonged to named individual scientists. Thus 1.1 % of individual Astro/Particle delegates were highly Twitter-active at conferences.

Of the 64 active tweeps identified from Other conferences, three (two news organisations and one individual) were retweeting from outside the conference; they were responsible for a total of 68 retweets. The remaining 61 highly active accounts were all conference attendees; this equates to 0.2 % of Other conference delegates. Of these 61 accounts, 21 were organizational in nature; 40 accounts belonged to named individual scientists. Thus 0.1 % of individual delegates were highly Twitter-active at Other conferences.

There is an order of magnitude difference in the percentage of Astro/Particle and Other conference delegates posting 10 or more tweets using the conference hashtag (1.1 vs 0.1 %).

A further analysis of the dataset showed that 11 delegates posted 100 or more tweets during a conference, and all 11 attended PACS90 conferences. These 11 Twitter accounts posted a total of 2225 tweets and retweets containing a conference hashtag, which is 25 % of the total number of tweets harvested in this study and is similar in magnitude to the entire Twitter output of the Other conferences.

This observation provokes the question of whether a small number of extremely active Twitter users might on their own generate the observed differences between Astro/Particle and Other conferences.

If one repeats the earlier analysis then, after removing all data pertaining to accounts that posted 10 or more tweets, the differences between Astro/Particle and Other conferences are indeed lessened. However, the differences are not entirely eliminated. For example, in terms of tweet rate, Astro/Particle accounted for 9 of the 10 most Twitter-active conferences. When all conferences are considered the median tweet rate for Astro/Particle conferences, excluding the most active users, falls to $28.3 _{6.1}^{74.3}$; however, this is still greater than the median tweet rate for Others of $1.1 _{0}^{1.7}$. The difference persists if, in addition to excluding highly active users, one also excludes conferences at which there was no Twitter activity. In this case, the median tweet rate for Others rises to $6.7 _{3.3}^{10.8}$ but the median tweet rate for Astro/Particle conferences remains higher at $28.7 _{18.4}^{113.3}$. Thus the small number of extremely active Twitter users does tend to skew the picture, but these users do not by themselves account for all the observed differences between Astro/Particle and Others.

The numbers of conferences within individual PACS areas are too small to make a statistical analysis worthwhile, but it is worth observing that none of the four PACS50 conferences (i.e. conferences devoted to the physics of gases, plasmas and electric discharges) yielded any tweets. The combined tweet rate for all conferences in each of the Other categories was rather consistent: 0.8 (PACS00), 0.3 (PACS20), 1.2 (PACS30), 1.1 (PACS40), 0 (PACS50), 1.3 (PACS60), 0.9 (PACS70) and 1.4 (PACS80). These rates are to be compared with combined tweet rates of 25.2 and 33.3 for PACS10 and PACS90 respectively. If one excludes those users who posted 10 or more tweets then the numbers change, but the conclusion is unaltered: tweet rates for PACS10 and PACS90 are an order of magnitude greater than for the rest of the classification scheme.

### Analysis of tweet content

Holmberg and Thelwall (2014) analysed differences in Twitter scholarly communication in five disciplines (astrophysics, biochemistry, digital humanities, economics and history of science) by selecting 1000 tweets for a bi-faceted content analysis. For Facet 1, Holmberg and Thelwall grouped the tweets into one of four types (*Retweets*; *Conversations*; *Links*; *Other*) while, for Facet 2, they grouped the tweets into four content categories (*Scholarly communication*; *Discipline*-*relevant*; *Not about science*; *Not clear*). The 8774 tweets harvested in the current work were subject to a similar analysis, but slight modifications to the Holmberg–Thelwall scheme were employed.

For Facet 1 designations, Holmberg–Thelwall adopted an essentially mechanical approach. The identification of tweets as *Retweets* was straightforward. *Conversations* were tweets that were not retweets and contained the @-sign as part of an @username. (In adopting this approach, Holmberg–Thelwall were following Honeycutt and Herring (2009), who identified that 90 % of tweets containing the @-sign were conversational in nature, and that 30 % of all tweets could be classified as conversational). *Links* contained tweets that were neither retweets nor conversations and contained a url. *Other* contained the remaining tweets. A preliminary analysis of the tweets in the present sample showed that the Holmberg–Thelwall Facet 1 dimensions were not mutually orthogonal: for example, if retweets are included, 38 % of tweets contained both an @ sign and a link. The Holmberg–Thelwall scheme was therefore slightly modified. Tweets were classified in type as being either *Original* or *Retweet*. An *Original* tweet was then further categorized as *Link* (if it contained a url) or *Conversation* (if it contained an @username). As explained above, some tweets could belong to both *Link* and *Conversation* categories.

The Holmberg–Thelwall Facet 2 dimensions of *Scholarly communication* and *Discipline*-*relevant* were inappropriate for the present study, given that all harvested tweets were by definition somehow related to scientific conference activity. A simpler scheme for classifying content was therefore adopted: *Retweets* were excluded and *Original* tweets were classified as being *Science*; *Non*-*science*; *Unclear*; *Non*-*English*. Tweets in the *Non*-*English* category were not further analysed; an analysis by a native speaker could, of course, place them in any of the other categories.

A typical example of a tweet classified as *Science* would be: “Margueron: Symmetry energy affects T, s (but not density) post bounce, but incompressibility parameter doesn’t change anything. #MICRA2015”. *Non*-*science* tweets were those referring to: general conference management; announcements from publishers or exhibitors; messages that focused on weather or the conference environment; those that attempted humour; the (many) that mentioned food and drink; and so on. A typical example of a tweet classified as *Non*-*science* would be: “@DSFD_Conference I heard a rumour of salmon… Quite excited! #DSFD2015”. A typical example from the *Unclear* category would be: “Like The Devil @ATLASexperiment #LeptonPhoton15”.

Table 5 contains data on tweet type for Astro/Particle and Other conferences. Compared to Others, a slightly lower proportion of Astro/Particle tweets are *Original*; an alternative way of expressing this is that a slightly higher proportion of Astro/Particle tweets were retweets. In Astro/Particle conferences, 30.8 % of original tweets were conversational in nature, as defined by inclusion of an @-sign. This figure is in agreement with previous studies (Honeycutt and Herring 2009; Boyd et al. 2010), which suggested that about 30 % of tweets are conversational in nature. A rather higher proportion of Other tweets were conversational: 43.6 %. Similarly, a greater proportion of Other tweets than Astro/Particle tweets contained links (60.1 vs 42.4 %).

**Table 5:** Type of tweet

	Astro/Particle	Others
% of *Original* tweets	43.7 %	55.4 %
	(2596 *Original* tweets)	(1569 *Original* tweets)
% *Link*	42.4 %	60.1 %
	(1100 of 2596 *Original* tweets)	(943 of 1569 *Original* tweets)
% *Conversation*	30.8 %	43.6 %
	(799 of 2596 *Original* tweets)	(684 of 1569 *Original* tweets)

Note that percentages need not sum to 100 %: some tweets are neither conversational nor contain a link, while some tweets are conversational in nature and also contain a link. If retweets are included, 34.7 % of Astro/Particle tweets had this dual nature; the figure for Others is 44.5 %

Table 6 contains data on the content of *Original* tweets. As can be seen, the language of tweets is overwhelmingly English. Although there is an inevitable element of subjectivity in classifying tweet content in this way, it seems clear that Astro/Particle tweets are more likely to focus on scientific issues than are tweets from Other conferences. Understanding the underlying source of this difference requires further research, but the observations mentioned above motivate two tentative suggestions that might be explored in more detail in a qualitative study. First, delegates at Other conferences appear to use Twitter in a more conversational manner, and are perhaps therefore more concerned in using the service for social uses, than those at Astro/Particle conferences. Second, as described in the “Twitter activity at conferences” section, Astro/Particle conferences are more likely to contain delegates that are extremely active Twitter users; if the motivation of these delegates is primarily to live tweet about the science being discussed in conference presentations then this would help explain the differences shown in Table 6.

**Table 6:** Content of tweets classified as *Original* (i.e. 2596 Astro/Particle tweets and 1569 Other tweets)

	Astro/Particle (%)	Other (%)
% of *Science* tweets	73.3	40.7
% of *Non*-*science* tweets	21.6	51.9
% of *Unclear* tweets	2.8	1.9
% of *Non*-*English* tweets	2.3	5.5

## Conclusion

The above analysis of 116 scientific conferences suggests that Twitter is used in a quantitatively and qualitatively different manner at conferences devoted to the physics of elementary particles and fields, and to geophysics, astronomy, and astrophysics, than at conferences in other fields of physics. The analysis showed that delegates at an Astro/Particle conference are four times more likely to be participating in an event where Twitter is used than are delegates at Other conferences. At conferences where Twitter is used, an Astro/Particle delegate is 4.5 times more likely to live tweet. The distribution of conference tweet rates (tweets per delegate per day) shows significant differences, with rates typically being higher at Astro/Particle conferences. If being highly Twitter-active at a conference is defined as posting 10 or more tweets from the event then an individual Astro/Particle delegate is 10 times more likely to be highly active than an individual delegate at Other conferences. Finally, tweets from Astro/Particle conferences are more likely to focus on science.

An obvious question arises: what might be the reason for the observed differences in Twitter use?

The data collected during current research is insufficient, by itself, to determine the origin of these differences. Nevertheless, a more detailed analysis of the highly active Twitter accounts suggests a possible explanation for the differences, which further qualitative research would be able to corroborate or discount.

As mentioned in the “Twitter activity at conferences” section above, there were a number of highly active Twitter accounts during conferences. Some belonged to organisations (conference organisers tweeting event information, research groups tweeting news, and so on) but the majority belonged to named individuals. In total, 45 delegates at Astro/Particle conferences and 40 delegates at Other conferences were highly active Twitter users. An analysis of the individual accounts highlighted a clear difference between the two populations.

Highly active accounts at Other conferences had a median number of followers of 243; this is entirely in line with the work of Darling et al. (2013), mentioned in “The Twitter platform” section, which found that the median number of followers of a sample of bioscientists was 241. On the other hand, highly active accounts at Astro/Particle conferences had almost double the median number of followers: 472. An examination of the online Twitter biographies of the 85 highly active users highlights a further substantial difference between the two groups: 47 % (21 from 45) of active Astro/Particle Twitter users explicitly mention some aspect of science outreach whereas for Others the number is only 5 % (2 from 40).

These figures give rise to the hypothesis that the observed difference in Twitter use at conferences is due to the different requirements of the two groups. As noted in the “Twitter use in scholarly scientific communication” section, the Twitter platform already meets a wide variety of use cases, so in this sense the suggestion is not surprising.

Particle physics and astrophysics are both examples of “big science”, with large multinational research teams and facilities that often have a dedicated press office. Both disciplines have a relatively high public profile. Within this environment public outreach is a well-recognised activity, and it may be that scientists in these disciplines view Twitter, along with other social media tools such as blogs, in addition to more traditional avenues, as another tool for outreach. This would be consistent with the finding that highly active Twitter users in these disciplines have a large median number of followers: their Twitter networks consist not just of professional scientists, but of lay people with an interest in these fields. It would also be consistent with the observation in the “Twitter activity at conferences” section that a relatively large number of non-scientists who did not attend a conference nevertheless retweeted content: these followers of Astro/Particle scientists would see conference tweets in their timelines. Furthermore, it offers an explanation as to why Astro/Particle tweets tend to focus on science: if a key driver for Twitter use is public outreach then it is natural that a proportion of tweets will focus on scientific topics.

For Twitter users in Other disciplines, where public outreach activity appears to be less ingrained, conference tweeting is used in a much more functional way: the focus is on social and practical topics regarding the conference. This is perhaps unsurprising since the 140-character limit imposed by Twitter makes an in-depth, peer-based discussion of scientific concepts extremely challenging. If the tool is deemed to be unsuitable for professional scientific communication, and is not widely used for public-facing and outreach activities, then its more social aspects become increasingly relevant.

Further qualitative research, broadening the scope to include “big science” fields in other areas of science, will be undertaken to test this hypothesis.

## Appendix

### List of conferences analysed

Tweets were analysed from the 116 conferences and meetings given below.

- 2nd Asia–Oceania Conference on Neutron Scattering.
- 2nd Conference on Heavy Ion Collisions in the LHC Era and Beyond.
- 2nd International Conference on Nanoscience and Nanotechnology.
- 3rd Annual Large Hadron Collider Physics Conference.
- 3rd Conference on Finite Dimensional Integrable Systems in Geometry and Mathematical Physics.
- 4th International Conference on New Frontiers in Physics.
- 4th International Symposium on Sensor Science.
- 5th European Conference on Molecular Magnetism.
- 5th International Conference on Collective Motion in Nuclei under Extreme Conditions.
- 5th International Conference on High Energy Density Physics.
- 6th Annual Symposium Physics of Cancer.
- 6th European Conference on Neutron Scattering.
- 6th International Conference on Advanced Nanomaterials.
- 6th International QCD@LHC Workshop.
- 6th International Symposium on Bifurcations and Instabilities in Fluid Dynamics.
- 7th International Scientific Conference on Physics and Control.
- 8th International Conference on Plasma Physics and Plasma Technology.
- 8th International Workshop on Top Quark Physics.
- 8th Workshop on Atomic and Molecular Physics.
- 9th International Conference on Inertial Fusion Sciences and Applications.
- 10th European Biophysics Conference.
- 10th IEEE Nanotechnology Materials and Devices Conference.
- 10th Principles and Applications of Control in Quantum Systems Workshop.
- 11th Conference on Lasers and Electro-Optics—Pacific Rim.
- 11th International Conference on Materials and Mechanisms of Superconductivity.
- 11th International Conference on the Properties and Applications of Dielectric Materials.
- 12th European Conference on Applied Superconductivity.
- 12th International Conference on Materials Chemistry.
- 12th International Symposium on Fusion Nuclear Technology.
- 12th US–Japan Seminar on Many Body Quantum Systems.
- 13th International Conference on Heavy Ion Accelerator Technology.
- 14th International Conference on Topics in Astroparticle and Underground Physics.
- 14th Marcel Grossmann Meeting—MG14.
- 14th RHESSI Workshop.
- 15th International Conference on Nanotechnology.
- 16th International Conference on X-ray Absorption Fine Structure.
- 16th International Small-Angle Scattering Conference.
- 17th International Conference on RF Superconductivity.
- 17th International Conference on the Strength Of Materials.
- 18th International Congress on Mathematical Physics 2015.
- 18th International Workshop on Computational Electronics.
- 19th Paris Cosmology Colloquium.
- 20th International Conference on Composite Materials.
- 20th International Workshop on Electromagnetic Nondestructive Evaluation.
- 20th International Workshop on Quantum Systems in Chemistry, Physics and Biology.
- 22nd International Colloquium on Magnetic Films and Surfaces.
- 22nd International Congress on Sound and Vibration.
- 24th International Conference on Discrete Simulation of Fluid Dynamics.
- 24th International Conference on the Numerical Simulation of Plasma.
- 24th International Materials Research Congress.
- 24th Nordic Rheology Conference.
- 25th Congress of International Society of Biomechanics.
- 27th International Symposium on Lepton Photon Interactions at High Energies.
- 28th International Conference on Defects in Semiconductors.
- 29th European Crystallographic Meeting.
- 29th International Conference on Photonic, Electronic and Atomic Collisions.
- 31st Panhellenic Conference on Solid State Physics and Materials Science.
- 33rd International Symposium on Lattice Field Theory.
- 35th Max Born Symposium: Planck Scale II.
- 37th International Free Electron Laser Conference.
- 45th European Solid-State Device Research Conference.
- 47th Annual Conference on Solid State Devices and Materials.
- 47th Conference of the European Group on Atomic Systems.
- 2015 European Nuclear Physics Conference.
- 2015 International Symposium on Quantum Fluids and Solids.
- 30 Years of Radioactive Ion Beam Physics.
- Accurate Astrophysics. Correct Cosmology.
- American Crystallographic Association 65th Annual Meeting.
- ASB6: The Origin, Distribution and Detection of Life in the Universe.
- Beyond Integrability: The Mathematics and Physics of Integrability.
- Bose–Einstein Condensation 2015.
- Concepts and Discovery in Quantum Matter.
- Concreep-10.
- Cosmo-15.
- Cosmology with CMB-S4.
- EPS HEP 2015.
- European Conference on Surface Science.
- European Congress and Exhibition on Advanced Materials and Processes.
- ExoMol 2015—Spectroscopy of Exoplanets.
- Exploring the Hot and Energetic Universe.
- Finite-Temperature Non-Equilibrium Superfluid Systems.
- Frontiers in Nanophotonics.
- Frontiers of Quantum and Mesoscopic Thermodynamics.
- Geometry and Physics of Spatial Random Systems.
- Greenhorn Meeting 2015.
- Heavy Ion Accelerator Symposium.
- Hot topics in General Relativity and Gravitation.
- Integrability in Algebra, Geometry and Physics: New Trends.
- International Beam Instrumentation Conference.
- International Conference Of Physics Students 2015.
- International Conference on Advanced Vibrational Spectroscopy.
- International Conference on Electron Spectroscopy and Structure.
- International Conference on Quantum Information Processing and Communication.
- IPEM Medical Physics and Engineering Conference.
- Karl Schwarzschild Meeting 2015.
- Logic, Relativity and Beyond.
- Low-x Meeting 2015.
- Mathematical Modelling and Computational Physics 2015.
- Metamaterials 2015.
- Microphysics in Computational Relativistic Astrophysics.
- Microscopy Conference 2015.
- Novel Quantum Materials and Systems.
- Pathways Towards Habitable Planets—II.
- Photonics in Switching 2015.
- Physical Properties Of Nanoparticles.
- Positive Grassmannians.
- Psi-k 2015 Conference.
- Quantum Astronomy and Stellar Imaging 2015.
- Quantum Physics and Logic.
- Reflections on the Atomic Nucleus.
- SDSS-IV Collaboration Meeting.
- Size-strain VII: Diffraction Analysis.
- Stochastic Analysis and Mathematical Physics 2015: the Biological Challenge.
- Theoretical and Observational Progress on Large-scale Structure of the Universe.
- Thermodynamics 2015.
- Trends in Nanotechnology International Conference.

## References

[CR1] about.twitter.com (2015). Twitter usage/company facts. Retrieved 5 October 2015.

[CR2] Bar-Ilan, J., Haustein, S., Peters, I., Priem, J., Shema, H., & J. Terliesner (2012). Beyond citations: Scholars’ visibility on the social web. In É. Archambault, Y. Gringras & V. Lariviére (Eds.). *Proceedings of the 17th international conference on science and technology indicators*. (pp. 98–109). Montréal: Science-Metrix et OST. Montréal, Quebec, September 5–8, 2012.

[CR3] Basner JE (2013). Measuring the evolution and output of cross-disciplinary collaborations within the NCI physical sciences-oncology centers network. Research Evaluation.

[CR4] Bik HM, Goldstein MC (2013). An introduction to social media for scientists. PLoS Biology.

[CR5] Botta F, Moat HS, Preis T (2015). Quantifying crowd size with mobile phone and Twitter data. Royal Society Open Science.

[CR6] Boyd, D., Golder, S., & Lotan, G. (2010). Tweet, tweet, retweet: Conversational aspects of retweeting on Twitter. In *Proceedings of the 43rd Hawaii international conference on system sciences*.

[CR7] Darling E, Shiffman D, Côté I, Drew J (2013). The role of Twitter in the life cycle of a scientific publication. Ideas in Ecology and Evolution.

[CR8] Earle PS, Bowden DC, Guy M (2011). Twitter earthquake detection: Earthquake monitoring in a social world. Annals of Geophysics.

[CR9] Gonzalez-Bailon S, Wang N, Rivero A, Borge-Holthoefer J, Moreno Y (2014). Assessing the bias in samples of large online networks. Social Networks.

[CR10] Holmberg K, Thelwall M (2014). Differences in Twitter scholarly communication. Scientometrics.

[CR11] Honeycutt, C., & Herring, C. (2009). Beyond microblogging: Conversation and collaboration via Twitter. In *Proceedings of the 42rd Hawaii international conference on system sciences.*

[CR12] Letierce, J., Passant, A., Breslin, J., & Decker, S. (2010). Understanding how Twitter is used to spread scientific messages. In *Proceedings of the WebSci10: Extending the frontiers of society on*-*line*, April 26–27, 2010, Raleigh, NC. Retrieved 16 October 2015 from http://journal.webscience.org/314/.

[CR13] Liu, Y., Kliman-Silver, C., & Mislove, A. (2014). The tweets they are a-changin’: Evolution of Twitter users and behavior. In: *Proceedings of the 8th international AAAI conference on weblogs and social media*. University of Michigan, 1–4 June 2014

[CR14] Ross C, Terras M, Warwick C, Welsh A (2010). Enabled backchannel: Conference Twitter use by digital humanists. Journal of Documentation.

[CR15] Scanfeld D, Scanfeld M, Larson EL (2010). Dissemination of health information through social networks: Twitter and antibiotics. American Journal of Infection Control.

[CR16] Van Noorden R (2014). Online collaboration: Scientists and the social network. Nature.

[CR17] Weller, K., Dröge, E., & Puschmann, C. (2011). Citation analysis in Twitter: Approaches for defining and measuring information flows within tweets during scientific conferences. In M. Rowe, M. Stankovic, A.-S. Dadzie & M. Hardey (Eds.). *Making sense of microposts* (#MSM2011), workshop at extended semantic web conference (ESWC’2011), Crete, Greece (pp. 1–12). CEUR workshop proceedings (Vol. 718). Retrieved 16 October 2015 from http://ceur-ws.org/Vol-718/paper_04.pdf.

[CR18] Weller, K., & Puschmann, C. (2011). Twitter for scientific communication: How can citations/references be identified and measured? In *Proceedings of the poster session at the web science conference 2011* (WebSci11), Koblenz, Germany. Retrieved 16 October 2015 from http://www.websci11.org/fileadmin/websci/Posters/153_paper.pdf.

[CR19] Williams, S. A., Terras, M., & Warwick, C. (2013). How Twitter is studied in the medical professions: A classification of Twitter papers indexed in PubMed. *Med 2.0 2013*; *2*(2):e2 Retrieved 16 December 2015 from www.medicine20.com/2013/2/e2. doi: 10.2196/med20.2269 10.2196/med20.2269 PMC4084770 25075237

[CR20] Woolston C (2015). Conference tweeting rule frustrates ecologists. Nature.

